# Screening and concurrent brief intervention of conjoint hazardous or harmful alcohol and tobacco use in hospital out-patients in Thailand: a randomized controlled trial

**DOI:** 10.1186/s13011-015-0018-1

**Published:** 2015-05-27

**Authors:** Supa Pengpid, Karl Peltzer, Apa Puckpinyo, Somchai Viripiromgool, Kriengsak Thamma-aphiphol, Kawinarat Suthisukhon, Dussanee Dumee, Thiprada Kongtapan

**Affiliations:** ASEAN Institute for Health Development, Mahidol University, Salaya Phutthamonthon, Nakhonpathom, Thailand; University of Limpopo, Turfloop Campus, Turfloop, South Africa; HIV/AIDS/STIs/and TB (HAST), Human Sciences Research Council, Pretoria, South Africa

**Keywords:** Conjoint alcohol and tobacco use, Brief intervention, Moderate risk, Tobacco use alcohol use, Hospital out-patients, Thailand

## Abstract

**Background:**

The aim of this study was to conduct a cluster randomized control trial to assess the efficacy of screening and brief intervention (SBI) for conjoint alcohol and tobacco use among hospital out-patients.

**Method:**

In all 620 hospital out-patients who screened positive for both tobacco and alcohol moderate risk in four hospitals were randomized into 2 control and 1 intervention condition using the hospital as a unit of randomization (2 intervention and 2 control hospitals) to 405 patients in the two control groups (tobacco only intervention, *n* = 199, and alcohol only intervention, *n* = 206) and 215 in the intervention group. The intervention or control consisted of three counselling sessions.

**Results:**

Results of the interaction (Group × Time) effects using GEE indicated that there were statistically significant differences between the three study groups over the 6-month follow-up on the ASSIST tobacco score (Wald χ^2^ = 8.43, *P* = 0.004), and past week tobacco use abstinence (Wald χ^2^ = 7.34, *P* = 0.007). Although there were no significant interaction effects on the other outcomes (Alcohol ASSIST score, low alcohol risk score, past week tobacco abstinence or low alcohol risk score, and past week tobacco abstinence and low alcohol risk score), the scores in all of the six outcome measures showed consistent improvements. For past week tobacco abstinence the tobacco only intervention was more effective than the alcohol only intervention and the integrated alcohol and tobacco intervention. For the outcome of low alcohol risk, the alcohol only intervention and the integrated alcohol and tobacco intervention was more effective than the tobacco only or alcohol only intervention.

**Conclusions:**

The study found that for past week tobacco abstinence the tobacco only intervention was more effective than the alcohol only intervention and the polydrug use (alcohol and tobacco) integrated intervention.

## Background

In a national population survey in Thailand the prevalence of joint (daily) smoking and harmful or hazardous alcohol consumption in adults over 14 years of age was among men 10.0 % [1], and in a large national study among high school students, Saingam *et al.* [2] found 10 % of boys and 3 % of girls were co-users of alcohol and tobacco. In different local studies in various health care settings in Thailand a high prevalence of alcohol and/or tobacco use was found, e.g., in primary care Southern Thailand past 3 months tobacco use was among any past 3 months substance user 91.3 % (of which 83.5 % were in the moderate risk group) and alcohol use 33.5 % (of which 43.2 % were in the moderate risk group) [3] and in general medical clinics 60 % had used alcohol and 73.9 % had used tobacco in the past three months [4]. International studies have shown that 20-30 % of patients who routinely present in primary care are hazardous or harmful drinkers [5].

In the Thai national health examination population survey the strongest predictor of harmful or hazardous alcohol consumption in both sexes was currently smoking, and likewise, the strongest predictor of current smoking is harmful or hazardous alcohol use [1]. There is a high association between nicotine and alcohol dependence [6]. Compared to one specific substance use dependence, conjoint nicotine and alcohol dependence are more severe and have a more unfavourable course [6]. Smoking increases during alcohol consumption [7], and heavy drinkers are less likely to attempt to quit smoking and are less likely to be successful when they make an attempt [8–10]. The integration of a brief intervention for tobacco use and problem drinking is important because of (1) the prevalence of problem drinking is higher among smokers than non-smokers [1, 11], (2) alcohol consumption has been identified as a trigger for smoking and for relapse to smoking among those who have already quit, and heavy drinkers have significantly lower rates of quitting smoking [11–13], and (3) the combined health risks of smoking and problem drinking are higher than the risks of smoking alone [11, 14]. Further, the existing literature [11, 15–19] on the concurrent treatment of smoking and drinking indicates that there may be some benefit to combining services for these addictive behaviours. Given this demonstrated overlap in smoking and drinking, the present study explores a brief intervention for conjoint alcohol problem and tobacco users using Kahler’s model [17].

To help reducing problems related to substance misuse, the World Health Organization (WHO) recommends the Alcohol, Smoking and Substance Involvement Screening Test (ASSIST) [20] and its linked brief intervention (BI) procedure to be used as an early intervention package in primary health care (PHC) settings [21–24]. Several meta-analyses have shown that screening using short questionnaires followed by brief intervention (comprising simple advice or psychological counselling) significantly reduces alcohol consumption in primary care populations [25–29]. Likewise, several meta-analyses found that physician advice for smoking cessation [30], brief opportunistic smoking cessation interventions [31] and combined pharmacotherapy and behavioural interventions for smoking cessation [32] are effective to increase abstinence in smoking. Further, the existing literature on the concurrent treatment of smoking and drinking indicates that there may be some benefit to combining services for these addictive behaviours [11, 15–19]. Compared with most other BI studies (e.g., alcohol brief intervention in Thailand: [33–38]), the present study combines two aspects that rarely are analyzed: a focus widened from a single substance to a brief integrative multi-substance (alcohol and tobacco) intervention and the efficacy of the inclusion of booster sessions for poly drug use [39]. There is lack of information on the importance and effects of an integrative brief intervention (focusing on both alcohol and tobacco use) of conjoint alcohol and tobacco users using Kahler’s model [17], compared to the single intervention of either smoking cessation or alcohol risk reduction of conjoint alcohol and tobacco users.

The aim of this study is to conduct a cluster randomized control trial to assess the efficacy of SBI for conjoint alcohol and tobacco use among hospital out-patients. Consenting patients screening positive for conjoint alcohol and tobacco use risk are randomized, with the hospital clinic being the unit of randomization into one of three arms: The first arm being a three sessions Brief Intervention for alcohol and tobacco arm (treatment arm) of conjoint alcohol and tobacco users using Kahler’s model [17], the second arm being the smoking cessation only of conjoint alcohol and tobacco users, and the third arm being the brief alcohol intervention only arm of conjoint alcohol and tobacco users.

## Methods

### Setting

The study setting is four district hospitals randomly selected from 8 district hospitals in Nakhon Patthom province in Thailand.

### Design

In order to assess the efficacy of the Screening and Brief Interventions (SBI) among participants found to be at moderate risk for alcohol and tobacco use, a cluster randomized controlled trial design was implemented. All out-patients were screened using the ASSIST. Patients who met the cut-off for moderate risk of conjoint alcohol and tobacco use, both in the intervention and control arms, were reassessed after baseline assessment at time 2 (3 months following intervention) and time 3 (6 months following intervention). The intervention comprised of three sessions including brief counselling about reducing alcohol use and smoking cessation. The trial incorporated cluster randomization of district hospitals to avoid the risk of contamination.

#### Study hypotheses

- Conjoint alcohol and tobacco users receiving the integrative (both alcohol and tobacco) brief intervention will experience a greater change in the mean number of ASSIST alcohol use risk scores and in the mean number of ASSIST tobacco use risk scores, compared with the individuals receiving either a tobacco use only or an alcohol use only intervention (control groups)

### Principles for recruitment

#### Inclusion/exclusion criteria

Patients, age between 18 and 60 years; able to participate in a 3- and 6-month follow-up post-intervention; able to give contact details for at least two to three other people (for purposes of follow-up); having a fixed address; not pending incarceration within the next 3 months; absence of cognitive impairment or severe behaviour problems; not intoxicated or going through withdrawal from alcohol or other drugs; and not currently in drug or alcohol or nicotine treatment. Participants who scored between 0 and 26 on the ASSIST for cannabis, cocaine, ATS and opioids, hallucinogens, sedatives, and inhalants, and participants who score low between 0–3 for tobacco and 0–10 for alcohol were excluded from enrolment into the study, but received information on drugs if relevant. Participants who scored between 4 and 26 (moderate risk) for tobacco and between 11–26 for alcohol (moderate risk) were enrolled in the study and randomised to either the Control or Intervention group. Participants who scored in the high risk category (27 or higher for any of the substances), or who had frequently injected drugs in the last three months (more than 4 times per month on average) were excluded from enrolment into the study and were referred to specialist substance use treatment services [37].

#### Randomisation

Randomisation was conducted using a secure remote randomization service. The 4 out of 8 district hospitals were randomly assigned to the treatment (co-joint intervention) and control arms (alcohol only or tobacco only interventions). Patients moderately at risk for conjoint alcohol and tobacco use were randomized to either the treatment or control groups. At clinic level all consecutive patients were systematically recruited over a period of 3–4 months.

#### Blinding

Participants (clinic staff members and patients) were not blind to their intervention status. However, to protect against information biases in the reporting of substance use, the data collection team who assessed the outcomes were blind to the hospital’s status as intervention.

### Procedure

Universal screening of all patients was used whereby all consecutive clients visiting the district hospital out-patient department were screened for substance use problems and in case of moderate conjoint alcohol and tobacco use risk were offered a brief counselling intervention. A health care provider informed the patient about the study and referred the patient for participation if interested. A research assistant (a professional researcher with a university degree, employed by the research institution) asked for permission/consent from patients attending the district hospital facility to participate in the stage 1 of the study, i.e., screening or baseline assessment using the ASSIST questionnaire. This took about 5 min. The research assistant was not involved in delivering the brief counselling intervention. All participants underwent the initial assessment and the research assistant scored the results of the ASSIST. Patients who scored between 4 and 26 on the ASSIST (moderate-risk range) for tobacco and scored between 11 and 26 on the ASSIST (moderate-risk range) for alcohol were then approached by the research assistant for a second informed consent for enrolment in stage 2, the intervention study. Patients scoring >26 on the ASSIST on any of the substances (including tobacco and alcohol) were referred for further management, and patients with tobacco use only scoring 0–26 and patients with alcohol use only scoring 0–26 were provided with a health education leaflet. For patients included in the study, the research assistant referred the patient to a trained research counsellor (the research counsellor has some professional training in counselling, with a university degree in a health related background) who carried out the intervention (experimental or control) of three sessions within a period of three weeks for all the participants after which they were followed up in face-to-face interviews at 3 months and 6 months following the intervention at the health facility, and assessments/interviews were done by the research assistant. Participants received in total 200 Baht transport reimbursement at 6-month follow-up. We received ethical approval from the Mahidol University Research and Ethics Committee (COA. No. 2014/111.1804). The hospital management of the study hospitals also provided approval for this study. The study was conducted from May 2014 to January 2015.

### Interventions

#### Brief Counselling (integrative for both alcohol and tobacco use) conjoint alcohol and tobacco users

The patients randomized and allocated to the intervention arm completed baseline measures and received three sessions of brief counselling for alcohol use reduction and tobacco use cessation intervention using the ASSIST-linked brief intervention for hazardous and harmful substance use manual for use in primary care [20, 21, 38]. In addition, Kahler *et al.*'s brief intervention for heavy drinking smokers was incooperated [17]: Feedback and discussion on the relationship between drinking and smoking, and on the potential effects of alcohol consumption on smoking cessation; an emphasis on personal responsibility for choosing to change one's behaviour; Advice to avoid or minimize drinking during the smoking cessation process; a Menu of options for carrying out a change strategy; use of Empathy by the clinician; and encouragement of Self-efficacy (i.e., confidence) for change.

#### Brief counselling (tobacco use only) conjoint alcohol and tobacco users

The patients randomized and allocated to this control arm received three sessions of brief counselling on tobacco use cessation using the ASSIST-linked brief intervention for hazardous and harmful substance use manual for use in primary care [20, 21, 38].

#### Brief counselling (alcohol use only) conjoint alcohol and tobacco users

The patients randomized and allocated to this control arm received three sessions of brief counselling for alcohol use reduction using the ASSIST-linked brief intervention for hazardous and harmful substance use manual for use in primary care [20, 21, 38].

### Counsellor training

The intervention counsellors consisted of trained research counsellors who delivered the interventions to patients as per usual clinic services in a private consultation room. All research counsellors who were suitable to deliver the brief counselling intervention received formal training (4 days) before data collection and supervision. The training took a practical, systems approach, aiming to facilitate the implementation of SBI in clinic operations rather than merely educating staff. The training curriculum contained modules addressing practical issues deemed essential to implementing the programme. For early identification of substance use problems in primary care the ASSIST [4] and for the brief intervention the WHO brief intervention package for the ASSIST-linked brief intervention for hazardous and harmful substance use were used [38]. In addition, Kahler *et al.*’s integrative module for alcohol and tobacco use was used [17]. To help protect against research counsellor drift, the brief intervention was completely manualized, including a Motivational Interviewing (MI) protocol and used to guide the research counsellor through the session content [40].

## Intervention quality assurance

Assessment quality control was overseen by project staff, and intervention fidelity was maintained by audio tape recording of intervention sessions, interventionist checklists and reviewed by Apa Puckpinyo who provided feedback to research counsellors. Process evaluation included a review of a random sample of 10 % of the assessments and intervention audiotapes by the study coordinator for fidelity to protocol. Supa Pengpid conducted quality assurance of 10 % of transcribed sessions and provided feedback to Apa Puckpinyo for the research counsellors. Monthly visits also included review, ongoing monitoring and quality assurance. Evaluation also included review of study data to ensure fidelity of the intervention in the hospital setting.

### Outcome measures

#### Patient measures

##### Baseline

All patients (i.e., those in the treatment and control arms) completed baseline measures which included a Demographic Questionnaire, the ASSIST [36], the timeline follow back (TLFB) interview (for alcohol and cigarette use and past 7 days) [41–44] and health seeking behaviour for substance use.

The primary outcome measure used in this study was the change in the ASSIST alcohol use scores and ASSIST tobacco use scores from baseline to follow-up. In addition, we examine the point prevalence tobacco use outcomes and drinking outcomes in terms of drinks per week from the Timeline Followback (TLFB) interview.

##### Follow-up

The same measures as detailed above were administered (at follow-up) to the intervention and control groups of patients at 3 months and again at 6 months following the intervention. To prevent lost to follow up, and retain participants in the program; the regular communication through series of communication via SMS and Letter/ Postcards for reminding that they were in the programme and was sent out at least 2 weeks prior interviewing date.

### Sample size calculation

The study assumes a 20 % reduction of the ASSIST score from previous studies [38, 45]. The sample size calculated by using PST Version 1.3, based on the average number of 40 patients per hospital per day, minimum difference detectable 20 %, standard deviation 0.6, at 95 % power and 5 % significant level, the minimum adjusted sample size was 468, and number for each cluster 156. It was expected that 10 % of participants may be lost prior to completing the 3-months and 6-months follow-up assessments so that the final sample was 515. To round up, a total of 600 alcohol and tobacco misuse patients were eligible and consented to be screened for substance misuse over a period of six months. Based on the pilot study in February 2014, found 20 % of target population have moderates risk for alcohol and tobacco. Thus, this project has to screen about 3000 patients to reach the sample size.

### Data analyses

Means, standard deviations, and percentages were used for descriptive statistics. Mann–Whitney *U* Test for continuous data and chi-square for categorical data were used to examine baseline differences between groups. We first inspected all outcome variables for distribution properties. Variables that were significantly skewed, the total ASSIST scores were transformed using the formula log10 (χ + 1) with non-transformed observed values presented in the table. The primary outcome was measured at three time points: baseline, three and at six months. If a participant dropped out, and was not present on the day of the interview or refused to answer questions the primary outcome at the end point of the trial was missing. The researchers used an intent-to-treat analysis by including all participants who completed baseline assessments and who were randomized to intervention conditions. Listwise deletion (i.e., using complete cases only) in the context of generalized estimating equations (GEE) was used with missing data. The extent of the missing component was 27.4 % at six months. The method used to take account of the stratified trial design, the repeated binary and linear nature of the primary outcomes (Risky drinking, Tobacco use abstinence) and the missing data at follow-up is a GEE approach [46]. Estimated treatment effects are reported with 95 % confidence intervals. IBM SPSS for Windows version 20.0 (SPSS, Inc., Chicago, IL, USA) was used for the calculations.

## Results

### Screening and randomization

**Fig. 1:**
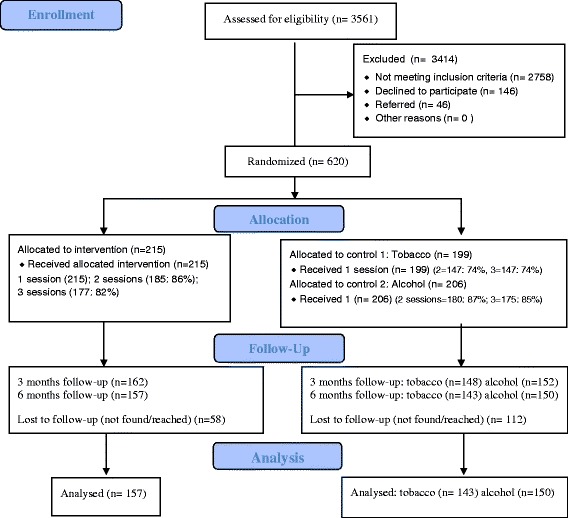
Flow-chart of participants in the trial

Figure 1 summarizes patient identification, recruitment, randomization, and follow-up numbers. We identified 3561 hospital out-patients, of which 2758 did not meet the inclusion criteria, 146 refused to participate, 46 were referred, resulting in 620 hospital out-patients who screened positive for both tobacco and alcohol moderate risk agreed to participate in the trial. Participants in the four hospitals were randomized into 2 control and 1 intervention condition using the hospital as a unit of randomization (2 intervention and 2 control hospitals) to 405 patients in the two control groups (tobacco only intervention, *n* = 199, and alcohol only intervention, *n* = 206) and 215 in the intervention group. The intervention consisted of three counselling sessions. In the integrated alcohol and tobacco intervention group 86 % attended two and 82 % three counselling sessions, in the tobacco control group 74 % attended two and three counselling sessions, and the alcohol control group 87 % attended two and 85 % all three counselling sessions. As illustrated in Fig. 1, at the 3-month follow-up, response rates for the intervention and two control groups were 75.3 %, 74.4 % and 73.8 %, respectively, and at 6 months, the intervention and two control groups response rates were 73.0 %, 71.9 % and 72.8 %, respectively. In the intervention group 30.2 % did not complete the last follow-up survey (i.e., the dropout rate was 30 %); in the two control groups, 28.1 % and 27.2 %, respectively, did not complete the last follow-up survey.

Attrition analyses were conducted to check for differential attrition by examining the condition by dropout interactions at baseline. The odds of completing follow-ups were not related to treatment condition. Dropout was significantly related to the ASSIST alcohol score (*t* = −2.22, df = 620; *P* = 0.027), indicating a relationship between this variable and dropout status depending on the ASSIST alcohol score: 16.1 in the dropout group versus 17.0 in the group that stayed on in the trial. Dropout was not related to condition (χ^2^ = 0.14, df = 3; *P* = 0.933), age (*t* = −0.48, df = 618; *P* = 0.632), sex (χ^2^ = 1.76, df = 1; *P* = 0.185), education (χ^2^ = 2.16, df = 2; *P* = 0.340), marital status (χ^2^ = 1.43, df = 2; *P* = 0.490), employment status (χ^2^ = 0.92, df = 1; *P* = 0.338), religion (χ^2^ = 1.18, df = 1; *P* = 0.553), ASSIST tobacco score (*t* = −0.41, df = 620; *P* = 0.685), tobacco units a week (*t* = 0.50, df = 620; *P* = 0.617), and alcohol units a week (*t* = 0.06, df = 620; *P* = 0.955).

### Participant characteristics

Table 1. summarizes sociodemographic and substance use characteristics of the study participants. Overall, the study sample was 97.7 % male, on average 33.6 (SD = 11.1) years of age, 53.5 % had primary or less education, 70.4 % were married or cohabiting, 99 % were Buddhist, 93.6 % were employed, and 67.6 % had 15000 or less Thai Baht monthly income.

**Table 1:** Baseline characteristics stratified by study condition (*N* = 620)

	Intervention (Tobacco plus alcohol) (*n* = 215)	Control (Tobacco) (*n* = 199)	Control (Alcohol) (*n* = 206)	Statistics	*P*-value
	N (%) or M (SD)	N (%) or M (SD)	N (%) or M (SD)	χ^2^ or F or *t*	
Sociodemographic variables					
Gender (N, % male)	210 (99.7)	191 (96.0)	205 (99.5)	5.74; df = 2	0.056
Age in years (M, SD) (range 18–60 years)	33.4 (10.8)	30.7 (10.9)	36.8 (11.0)	15.74; df = 2	<0.001
Education					
Primary or less	112 (52.3)	108 (54.8)	111 (53.9)	2.56; df = 4	0.690
Secondary	93 (43.5)	77 (39.1)	80 (38.8)		
Postsecondary	9 (4.2)	12 (6.1)	15 (7.3)		
Marital status					
Never married	60 (28.0)	46 (23.4)	48 (23.3)	6.47; df = 4	0.126
Married or cohabiting	140 (65.4)	146 (74.1)	150 (72.8)		
Separated/Divorced/Widowed	14 (6.5)	5 (2.5)	8 (3.9)		
Religious affiliation: Buddhist (vs. others)	213 (99.1)	196 (98.5)	205 (99.5)	2.17; df = 2	0.711
Employed (vs. unemployed or other)	205 (95.8)	176 (89.3)	196 (95.6)	9.16; df = 2	0.011
Monthly income					
<10000 Baht	67 (31.3)	58 (29.4)	45 (22.0)	6.78; df = 4	0.164
10000-15000 Baht	84 (39.3)	71 (36.0)	93 (45.4)		
>15000 Baht	63 (29.4)	68 (34.6)	67 (32.7)		
Tobacco and alcohol use					
Tobacco use (ASSIST score) (M,SD)	19.2 (4.4)	21.9 (3.6)	19.3 (4.4)	27.77; df = 2	<0.001
Alcohol use (ASSIST score) (M,SD)	16.9 (4.6)	16.2 (4.3)	17.2 (3.9)	2.86; df = 2	0.050
Past week tobacco use (units)	78 (52)	66 (49)	70 (48)	3.58; df = 2	0.032
Past week alcohol use (units)	22 (27)	19 (22)	21 (22)	1.03; df = 2	0.350
Other substance use (any frequency in the past 3 months)					
Cannabis	1 (0.5)	1 (0.5)	1 (0.5)		
Cocaine	0	0	0		
Amphetamine	0	1 (0.5)	0		
Solvents	0	0	0		
Diazepam or sleeping pills	2 (0.9)	0	0		
Hallucinogens	0	0	0		
Opium	0	0	0		
Other addictive substances	1 (0.5)	0	0		

*M* Mean; *SD* Standard Deviation; *df* degree of freedom; χ^*2*^ Chi-square

With regard to substance use variables, the overall mean ASSIST score was for tobacco use 20.1 (SD = 4.3) and for alcohol use 16.8 (SD = 4.3). Using the Timeline Followback (TLFB) interview the mean number of tobacco use units (cigarettes) was 71.7 (SD = 50.5) in the past 7 days and 21.0 (SD = 24.6) alcohol standard drinks in the past 7 days. Less than 1 % of the participants reported the use of other substances, other than alcohol and tobacco (see Table 1.). In terms of health care seeking behaviour for tobacco use problems in the past 3 months, 1.1 % had consulted a health facility, 0.3 % a traditional or complementary medicine provider, 2.6 % self-help or quit line, and 1 % other providers. Regarding health care seeking behaviour for alcohol use problems in the past 3 months, 0.2 % had consulted a traditional or complementary medicine provider and 0.7 % other providers. Almost two-thirds of participants (65.8 %) indicated that most of the time or every time they would smoke (or use tobacco products) more whenever they are engaged in drinking alcohol (see Table 2).

**Table 2:** Health care seeking for tobacco and or alcohol use and tobacco use drinking interaction stratified by study condition (*N* = 620)

	Intervention (Tobacco plus alcohol) (*n* = 215)	Control (Tobacco) (*n* = 199)	Control (Alcohol) (*n* = 206)	Statistics	
	N (%) or M (SD)	N (%) or M (SD)	N (%) or M (SD)	χ^2^	*P*-value
In the past three months did you consult for your tobacco use any health care provider					
Health facility	4 (1.9)	1 (0.5)	2 (1.0)	1.83; df = 2	0.397
Traditional/Complementary medicine provider	2 (0.9)	0	0	3.83; df = 2	0.146
Self-help or quit line	8 (3.8)	6 (3.0)	2 (1.0)	3.48; df = 2	0.173
Other provider	2 (1.0)	2 (1.0)	2 (1.0)	.001; df = 2	0.999
In the past three months did you consult for your alcohol use any health care provider					
Health facility	0	0	0		
Traditional/Complementary medicine provider	1 (0.5)	0	0		
Self-help or quit line	0	0	0		
Other provider	2 (1.0)	1 (0.5)	1 (0.5)		
Tendency of smoking more when drinking					
Never	33 (15.5)	34 (17.3)	43 (21.0)	192.36;	<0.001
Sometimes	10 (4.7)	17 (8.7)	37 (18.0)	df = 10	
Half of the time	5 (2.3)	8 (4.1)	23 (11.2)		
Most of the time	58 (27.2)	15 (7.7)	95 (46.3)		
Every time	107 (50.2)	122 (62.2)	7 (3.4)		

### Main treatment effects

Results of the interaction (Group × Time) effects using GEE indicated that there were statistically significant differences between the three study groups over the 6-month follow-up on the ASSIST tobacco score (Wald χ2 = 8.43, *P* = 0.004), and past week tobacco use abstinence (Wald χ2 = 7.34, *P* = 0.007). Although there were no significant interaction effects on the other outcomes (Alcohol ASSIST score, low alcohol risk score, past week tobacco abstinence or low alcohol risk score, and past week tobacco abstinence and low alcohol risk score), the scores in all of the six outcome measures showed consistent improvements (see Table 3).

**Table 3:** Alcohol and tobacco -related outcome measures at baseline, 3-month and 6-month follow-up

		Intervention	Control	Control	GEE (Time)		GEE (Group x Time)	
Variables	Time	Alcohol and Tobacco	Tobacco only	Alcohol only	Wald Chi-square	*P*-value	Wald Chi-square	*P*-value
Criterion variables								
Past week tobacco use units (M, SD)	Baseline (n = 620)	78 (52)	66 (49)	70 (48)	332.38	<0.001	2.60	0.107
	3 months (n = 462)	41 (38)	22 (32)	36 (37)				
	6 months (n = 450)	40 (35)	20 (29)	37 (42)				
Past week alcoholic use units (M, SD)	Baseline (n = 620)	22 (27)	19 (22)	21 (22)	206.48	<0.001	3.01	0.083
	3 months (n = 462)	8 (15)	5 (7)	7 (12)				
	6 months (n = 450)	12 (18)	5 (9)	6 (10)				
Tobacco ASSIST total (M, SD)	Baseline (n = 620)	19.2 (4.4)	21.9 (3.6)	19.3 (4.4)	485.08	<0.001	8.43	0.004
	3 months (n = 462)	7.0 (4.9)	9.1 (9.1)	8.9 (6.7)				
	6 months (n = 450)	6.3 (3.9)	9.1 (9.4)	8.5 (6.5)				
Past week tobacco abstinent (N, %)	Baseline (n = 620)	0 (0.0)	0 (0.0)	0 (0.0)	2000.16	<0.001	7.34	0.007
	3 months (n = 462)	31 (19.1)	61 (41.2)	37 (24.3)				
	6 months (n = 450)	28 (17.7)	70 (49.0)	40 (26.7)				
Alcohol ASSIST total (M, SD)	Baseline (n = 620)	16.9 (4.6)	16.2 (4.3)	17.2 (3.9)	1810.97	<0.001	1.80	0.179
	3 months (n = 462)	5.6 (5.6)	7.5 (7.4)	6.3 (6.3)				
	6 months (n = 450)	6.1 (4.9)	8.6 (8.0)	4.8 (5.7)				
Alcohol ASSIST score (<11) (N,%)	Baseline (n = 620)	0 (0.0)	0 (0.0)	0 (0.0)	486.58	<0.001	1.07	0.301
	3 months (n = 462)	131 (80.9)	96 (64.9)	110 (72.4)				
	6 months (n = 450)	127 (80.9)	86 (60.1)	123 (82.0)				
Past week tobacco abstinent OR Alcohol ASSIST (<11) (N,%)	Baseline (n = 620)	0 (0.0)	0 (0.0)	0 (0.0)	745.64	<0.001	1.05	0.305
	3 months (n = 462)	102 (63.0)	63 (42.6)	87 (57.6)				
	6 months (n = 450)	101 (64.3)	54 (37.8)	91 (60.7)				
Past week tobacco abstinent AND Alcohol ASSIST (<11) (N,%)	Baseline (n = 620)	0 (0.0)	0 (0.0)	0 (0.0)	737.40	<0.001	0.88	0.348
	3 months (n = 462)	30 (18.5)	47 (31.8)	30 (19.6)				
	6 months (n = 450)	27 (17.2)	51 (35.7)	36 (24.0)				

For past week tobacco abstinence the tobacco only intervention was more effective than the alcohol only intervention and the integrated alcohol and tobacco intervention (see Table 4).

**Table 4:** Treatment effect by intervention type

Outcome	Group x Time-Intervention	B (CI 95 %)	*P*-value
Past week tobacco abstinent	Alcohol + Tobacco versus Tobacco only	−1.34 (−1.74, −0.94)	<0.001
	Alcohol + Tobacco versus Alcohol only	−0.50 (−0.94, −0.06)	0.028
	Tobacco only versus Alcohol only	0.84 (0.47, 1.21)	<0.001

*B* Beta Coefficient; *CI* Confidence Interval

## Discussion

To our knowledge, this is the first randomized trial to evaluate the efficacy of a concurrent brief intervention of conjoint moderate risk of alcohol and tobacco use, compared to the single intervention of either smoking cessation or alcohol risk reduction of conjoint alcohol and tobacco users, in hospital out-patients in Thailand. The trial was conducted in 4 district hospitals, with out-patients in two hospitals serving as intervention group, the third hospital with the single tobacco use cessation serving as control group one and the fourth hospital with the single alcohol intervention serving as control group two. Self-reported outcome data suggest that the all three intervention approaches, the provision conjoint alcohol and tobacco intervention and the single tobacco or alcohol control intervention were all effective in reducing levels of moderate risk of alcohol and or tobacco use in hospital out-patients. From baseline to 3- and 6-month follow-up, both alcohol consumption and tobacco use declined significantly in both intervention (conjoint intervention) and control groups (single intervention). Contrary to a previous review [47], this study found a significant reduction of tobacco use in the alcohol use intervention control group, meaning that a brief alcohol intervention also reduced cigarette smoking. It is possible that there is a spill over effect when tackling one risk behaviour it could also reduce another risk behaviour.

The alcohol outcomes were reasonably good, and comparable to those reported in previous studies with hospital patients [48, 49], and the self-reported past 7 days tobacco use abstinence was also good (with the lowest, 17.7 %, in the integrated intervention and the highest, 49.0 %, in the single tobacco use intervention), compared to previous studies [50, 51].

Results of the interaction (Group × Time) effects using GEE indicated that there were statistically significant differences between the three study groups over the 6-month follow-up on the ASSIST tobacco score and past week tobacco use abstinence. We would have expected that participants receiving the integrated or concurrent alcohol and tobacco use intervention were significantly more likely to be abstinent from tobacco use than those receiving a tobacco use only intervention, as found in a previous study [17]. However, this study found that the tobacco use only intervention was more effective in achieving tobacco use abstinence than the integrated or concurrent alcohol and tobacco intervention. This finding is unclear and needs further investigation.

### Study limitations

Our study has several limitations, including the loss of patients at each follow-up point.

Further, alcohol and tobacco use was only assessed by self-report and should be verified by biomedical measures in future studies [17]. Moreover, the study only assessed short-term intervention effects (6 months) and longer term assessments (12 months) would be needed to assess the sustainability of intervention effects. Moreover, the study included volunteering out-patients from only one or two district hospitals per intervention arm, and thus not meaningful clusters could be formed for randomization and analysis. Further, the study found a large proportion (27 %) were lost to follow up at 6 months, while in the estimation of the sample size the lost to follow-up was set at 10 %, thus, the dataset obtained is incomplete and may not be representative of the groups. Moreover, dropout was associated with a low ASSIST alcohol score. Since the first ASSIST measurements were disclosed to the groups those not considering themselves at higher risk were less motivated to continue in the study. Another study limitation included that only a single blind method of the observers has been included in the methodology, while a double or triple blind is preferred. This being so, may have influenced results especially in control groups receiving only alcohol or only tobacco counselling.

## Conclusion

The study found that for past week tobacco abstinence the tobacco only intervention was more effective than the alcohol only intervention and the polydrug use (alcohol and tobacco) integrated intervention. Further, research is needed to show if a polydrug use (alcohol and tobacco) integrated intervention can be more effective than a single drug use intervention. The ASSIST brief intervention recommend, “While feedback would be given on all substances scoring in the moderate or high risk range, the focus of the intervention should be directed toward the substance(s) that are creating the most problems for the client or is of most concern to the client.”([38], p.26)
